# Editorial: The interplay between endocrine and immune systems in metabolic diseases

**DOI:** 10.3389/fendo.2024.1385271

**Published:** 2024-03-14

**Authors:** Lan Xiao, Weihao Wang, Pingping Han

**Affiliations:** ^1^ School of Medicine and Dentistry, Griffith University, Gold Coast, QLD, Australia; ^2^ Department of Endocrinology, Beijing Hospital, National Center of Gerontology, Institute of Geriatric Medicine, Chinese Academy of Medical Sciences, Beijing, China; ^3^ School of Dentistry, University of Queensland, Brisbane, QLD, Australia

**Keywords:** metabolic diseases, diabetes, immunomodulation, immune-endocrine crosstalk, macrophages

Currently, the management of metabolic diseases remains one of the major clinical challenges, and the accumulation of patients with metabolic diseases has caused significant public health burdens worldwide in recent years. Metabolic diseases are a group of diseases associated with metabolic dysregulation in various tissues/organs, such as obesity, diabetes, hereditary hemochromatosis, cystic fibrosis, etc. (1, 2). The pathogenesis of metabolic diseases is largely unknown, which includes a complex of genetic drivers and overlapping molecular pathways. Recent investigations suggest that the metabolic diseases are associated with immune system dysfunctions, mostly appear as chronical inflammation, which is due to eccrinology system to result in abnormal metabolism in both systemic levels and local tissues (3–5). Specifically, the metabolic changes (e.g., energy metabolism, glucose metabolism, lipid metabolism, etc.) have been found to directly regulate immune cell differentiation, function, polarization, etc. (3–5), whereas the detailed cellular and molecular mechanisms under these phenomena are not clear yet, suggesting a significant knowledge gap in endocrinology.

The aim of this Research Topic was to investigate the interplay between endocrine and immune systems in the pathogenesis of metabolic diseases, especially to understand the interplay between cells from endocrine and immune systems, how the interplay leads to metabolic changes on immune cells, how these metabolic changes affect the function/differentiation/polarization of immune cells, how these abnormal immune cells result in the pathogenesis of metabolic diseases, and the underlying molecular mechanisms. The Research Topic currently includes six papers, including three basic-science research papers on understanding the role of immune cells in thyroid diseases and diabetes at single-cell level, two clinical research papers on potential application of immune-regulatory natural component on diabetes prevention. These papers cover multidisciplinary research from the fields of immunology, endocrinology, and biotechnology.

In the paper “*Single-cell RNA sequencing reveals the role of cell heterogeneity in the sex difference in primary hyperparathyroidism*” (Lu et al.) and paper “*Autoimmune thyroid disease disrupts immune homeostasis in the endometrium of unexplained infertility women—a single-cell RNA transcriptome study during the implantation window*” (Xie et al.), single-cell RNA sequencing has been utilized to investigate the immune microenvironment for autoimmune thyroid disease (ASD) and primary hyperparathyroidism (PHPT), respectively. In comparison to individuals without ASD, those with ASD exhibited elevated levels of T CD4^+^, cNK, ILC3, T CD8^+^ GZMK^+^, T CD8^+^ Cytotoxic, and ILC3 CD3E- cells within the CD45^+^ immune cell populations of endometrium samples obtained from unexplained infertile women. This observation suggests a potential link between immune imbalance and embryo implantation issues. Further subcluster analysis of parathyroid cells from PHPT patients revealed that SPARCL1-OC and ISG15-OC are prevalent in females, whereas males exhibit higher levels of S100A13-PCC and PTHLH-OC. These findings provide insights into the gender-specific distribution of certain cell populations within parathyroid tissues (Lu et al.). The biotechnology for single-cell level analysis was also applied in diabetic investigation. In the paper “*Mass cytometry reveals the corneal immune cell changes at single cell level in diabetic mice*”, mass cytometry by time of flight (CyTOF) was adopted to examine the alterations of immune cell clusters in diabetic mice with corneal disorders (Qin et al.). In this study, CyTOF allowed for the comparison of corneal cells harvested from diabetic and non-diabetic mice at the single cell level. The CyTOF analysis showed eight main immune cell subsets: CD8^+^T cells, CD4^+^ T cells, γδ T cells, innate lymphoid cells (ILCs), myeloid-derived suppressor cells (MDSCs), dendritic cells (DCs), macrophages and monocytes. Diabetes lead to significant immune microenvironment alterations in corneal tissue, characterised as decreased CD103^+^CD8^+^T_RM_ cells and regulatory T cells (Tregs) and increased γδ T cells and CD11b^+^Ly6G^+^MDSCs. These dysregulated immune cell composition and function in corneal may lead to corneal tissue damage or impair tissue repair, whereas further investigations are required to find out the different roles of CD103+CD8+TRM cells, Tregs, γδ T cells and CD11b^+^Ly6G^+^MDSCs in the pathogenesis of diabetic corneal complications. Taken together, these three studies suggest the biotechnique for single-cell level analysis can serve as efficient tools for investigating the interplay between endocrine and immune systems in the pathogenesis of metabolic diseases, which can be employed in future studies.

In the clinical research paper “*Safety and efficacy of immune checkpoint inhibitor cancer therapy in patients with preexisting type 1diabetes mellitus*”, a retrospective, case-controlled multicenter study compared adult patients with or without type 1 diabetes mellitus (T1DM) who received one or more FDA-approved immune checkpoint inhibitor (ICI) therapies for malignancies (Hilder et al.). ICI therapy is an emerging tool in cancer treatment by facilitating the host immune cells to target cancer cells, however its clinical application is limited by the frequent (in 60% of patients) development of immune-related adverse events (IRAEs) which usually affects the endocrine system. The results in this paper showed that the incidence and severity of all grade IRAEs were similar in patients with preexisting T1DM compared with matched controls without T1DM, as well as the effect of ICI on tumour control and overall survival rate. These results suggest that a preexisting T1DM would not affect the ICI monotherapy, and ICI can be applied in cancer immunotherapy in T1DM patients. In the clinical research paper “*A self-controlled, cross-over study of intensive insulin treatment with needle-based injection versus needle-free injection in hospitalized patients with type 2 diabetes*” (Wu et al.), a self-control study was performed to compare the therapeutical effects of needle-free injection device (using needle-free syringe) and needle injection device in the intensive treatment of type 2 diabetes mellitus (T2DM) patients. Results showed that the needle-free system can improve glycemic control and fluctuation. Furthermore, the needle-free system can reduce the dose of short-acting insulin and relieve the painful experience. As a new injection technology, needle-free injection may become an alternative way for diabetic patients in the future.

In the review paper “*How do parasitic worms prevent diabetes? An exploration of their influence on macrophage and β-cell crosstalk*”, the authors thoroughly summarized the interplay between macrophages and insulin-producer pancreatic β cells and in the pathogenesis of T1DM and T2DM, and discussed how helminths regulates macrophage-β cell crosstalk to prevent diabetes (Camaya et al.). In T1DM or T2DM, β cells either serve as a source of auto- and neo-antigens or produce pro-inflammatory factors, respectively, to activate the antigen presenting cells (e.g., macrophages) and then T cells to trigger inflammatory immune response. The inflammatory environment eventually leads to β cell death and diabetes. The preventive effect of helminth in diabetes arises from its immunomodulatory capacity. Especially for the macrophage-β cell crosstalk, helminth can enhance the polarization of anti-inflammatory M2 phenotype and therefore minimize excessive macrophage inflammatory response; this consequently reduce the damage on β cells and prevent their exhaustion/death. Furthermore, helminth infection can remodel the host systemic metabolism by producing molecules to interact with β cells and modulating the gut-islet axis, which eventually leads to the shift in metabolic pathways in the immune system to regulate host immune inflammatory response and therefore, modulate the immune cell-β cell crosstalk to prevent both T1DM and T2DM. This review paper further explained the importance of immune-endocrine system crosstalk in the pathogenesis of metabolic diseases such as diabetes, suggesting that this crosstalk can serve as efficient therapeutical targets in diabetes treatment. It also suggests that helminth derived bioactive components could be considered in the development of therapeutical approaches for diabetes.

It is expected that through this Research Topic, a deeper understanding and new knowledge on the pathogenesis of metabolic diseases will be achieved. In addition, potential therapeutical approaches to target the endocrine-immune interplay are proposed, which can shed lights on the treatment of metabolic diseases in the future. Especially, potential therapeutical approaches to target the endocrine-immune interplay are under the scope, which aim to provide therapeutic candidates for metabolic diseases in the future. The preferred specific sub-themes include the cellular and molecular mechanisms under the endocrine-immune interplay, the immunometabolism, and therapeutic strategies/approaches to target the endocrine-immune interplay. The types of manuscripts includes research paper, review, and mini review.

Overall, this Research Topic recruited novel insights from both basic science and clinical investigations regarding the pathogenesis and potential therapeutical approach against metabolic diseases. These studies are expected to benefit metabolic disease research and treatment in the future.

## Author contributions

LX: Writing – original draft, Writing – review & editing. WW: Writing – original draft. PH: Writing – original draft.
